# Gender-specific disease trajectories prior to the onset of COPD allow individualized screening and early intervention

**DOI:** 10.1371/journal.pone.0288237

**Published:** 2023-07-07

**Authors:** Michelle Hagmann, Florent Baty, Frank Rassouli, Micha T. Maeder, Martin H. Brutsche

**Affiliations:** 1 Lung Center, Cantonal Hospital St. Gallen, St. Gallen, Switzerland; 2 Department of Cardiology, Cantonal Hospital St. Gallen, St. Gallen, Switzerland; University of Copenhagen: Kobenhavns Universitet, DENMARK

## Abstract

**Background:**

Nation-wide hospitalization databases include diagnostic information at the level of an entire population over an extended period of time. Comorbidity network and early disease development can be unveiled. Chronic obstructive pulmonary disease (COPD) is an underdiagnosed condition for which it is crucial to identify early disease indicators. The identification of gender-specific conditions preceding the onset of COPD may reveal disease progression patterns allowing for early diagnosis and intervention. The objective of the study was to investigate the antecedent hospitalization history of patients newly diagnosed with COPD and to retrace a gender-specific trajectory of coded entities prior to the onset of COPD.

**Material and methods:**

A population-wide hospitalization database including information about all hospitalizations in Switzerland between 2002 and 2018 was used. COPD cases were extracted from the database and comorbidities occurring prior to the onset of COPD identified. Comorbidities significantly over-represented in COPD compared with a 1:1, age- and sex-matched control population were identified and their longitudinal evolution was analyzed.

**Results:**

Between 2002 and 2018, 697,714 hospitalizations with coded COPD were recorded in Switzerland. Sixty-two diagnoses were significantly over-represented before onset of COPD. These preceding comorbidities included both well-established conditions and novel links to COPD. Early pre-conditions included nicotine and alcohol abuse, obesity and cardiovascular diseases. Later comorbidities included atrial fibrillation, diseases of the genitourinary system and pneumonia. Atherosclerotic heart diseases were more prevalent in males, whereas hypothyroidism, varicose and intestinal disorders were more frequent in females. Disease trajectories were validated using an independent data set.

**Conclusions:**

Gender-specific disease trajectories highlight early indicators and pathogenetic links between COPD and antecedent diseases and could allow for early detection and intervention.

## Introduction

Chronic obstructive pulmonary disease (COPD) is a common, preventable and treatable but incurable lung disease. COPD causes a significant economic burden primarily due to hospital admissions [1–6]. In the European Union the costs associated with COPD are estimated to be about 40 billion euros, i.e. 3.4% of the total healthcare spending. The estimated worldwide prevalence for COPD is 7.6% [7], but varies from study to study [8–11]. Apart from the economic burden, COPD also causes a social burden, increases morbidity and mortality for the patient [3, 5, 12]. According to the World Health Organization it is the third leading cause of death with more than 3 million people dying from COPD each year.

Several risk factors can contribute to the development of COPD. Studies have shown that the primary cause of COPD is exposure to tobacco smoke, followed by other risk factors such as indoor or outdoor air pollution as well as occupational dust and fumes [12–16]. In developed countries the prevalence of COPD is often directly related to the prevalence of smoking [1], whereas in more rural areas of developing countries exposure to biomass fumes also needs to be taken into account [17, 18]. Apart from the exposure to these noxae, there are also genetic characteristics and early life events involved in the pathogenesis of COPD [19, 20]. In a study investigating early life origins of COPD, Svanes and colleagues reported that “the impact of childhood disadvantage was as large as that of heavy smoking” [19]. Lange and collaborators suggested that a substantial proportion of persons who develop COPD had a low forced expiratory volume in 1 second (FEV_1_) level in early adulthood [20]. COPD is often associated with clusters of comorbidities, which can worsen the outcome of patients [21]. Data from several studies showed that co-existing comorbidities affect mortality strongly [8, 9, 22]. It is now well established that a significant number of patients with COPD die from cardiovascular diseases especially in the early disease stages.

Gender is also an important factor to take into account in the development of COPD [23]. The pathophysiology of COPD is influenced by gender-specific differences [24]. Studies show that women are more susceptible to tobacco smoke [25]. There is more and more evidence showing that gender-specific approaches to COPD are crucial.

If diagnosed and treated early, the progression of COPD can be decelerated [12, 13]. Consequently, it is crucial to prevent COPD effectively and find strategies to detect and treat it at an early stage. Notwithstanding, many patients with COPD remain undiagnosed in the early stages of the disease [6, 7, 26–28]. In another study focusing on the early detection of COPD, Vandervoorde and colleagues found that underdiagnosis of COPD was more frequent in the younger age categories [29].

Apart from classical screening, there is a growing body of literature that recognizes the importance of disease associations, multimorbidity and temporal disease trajectories [30]. Along these lines, Jensen et al. used registry data of 6.2 million patients to descry temporal disease trajectories [31], which they claim to be useful for predicting and preventing future diseases of individual patients.

Thus, analyzing antecedent hospitalizations occurring prior to the first-time diagnosis of COPD could improve the understanding on how the disease presents itself in an early stage and could reveal key factors that are associated with a future onset of COPD. Using a nation-wide hospitalization database, the aim of the current study was to investigate the nature and the time evolution of the comorbidities prior to the onset of COPD in both genders, assuming that this can unveil patterns that help diagnosing COPD before it is clinically manifest.

## Materials and methods

### Nation-wide hospitalization database

In-patient data were extracted from a hospitalization database provided by the Swiss Federal Office for Statistics. The database offers a nation-wide coverage of all hospitalizations in Switzerland between 2002 and 2018 (17 years). Patient information was fully anonymized and no written informed consent was required. For research purposes the Swiss Federal Office for Statistics provides regulated access to the data. No ethical approval was required for the retrospective analysis of this data set.

All diagnoses were coded using the German modification of the International Classification of Disease version 10 (ICD-10-GM). The ICD-10-GM coding system assigns diseases a hierarchical code including one letter and up to four digits providing increasing details on the disease. The database included one main diagnosis and up to 50 additional co-diagnoses.

The database included 24’239’724 hospitalization entries in the period between 2002 and 2018. Every patient had a unique anonymous identifier which could be tracked over the observation period. Information including the year and month of hospitalization, the patient’s age (5-year range) and gender, the length of hospital stay and in-hospital mortality, as well as the patient’s region of residence and the canton of the institution were available.

The data set was imported into an SQL database (SQLite version 3.31.1) and interfaced with the R statistical software using the dedicated package RSQLite.

### COPD cases and nested case control design

The disease trajectories of patients with COPD were investigated using the following strategy. First, all hospitalization cases including a (co-)diagnosis of COPD (ICD-10-GM code J44*) were extracted from the database. The ICD-10-GM code J44* included the subcategories J44.0 (Chronic obstructive pulmonary disease with acute lower respiratory infection), J44.1 (Chronic obstructive pulmonary disease with acute exacerbation, unspecified), J44.8 (Other specified chronic obstructive pulmonary disease) and J44.9 (Chronic obstructive pulmonary disease, unspecified). The terminology of COPD always referred to the same disease entity and its coding stayed unchanged throughout the time framework of the current study (2002–2018). Unique patient identifiers were used to extract all hospitalizations preceding the onset of COPD.

A control population including hospitalized patients who were never diagnosed with COPD was extracted from the database using a random extraction procedure. This 1:1 nested-control population was matched for age, gender and month of hospitalization.

### Statistical considerations

Patient’s baseline characteristics were summarized using descriptive statistics. Fisher’s exact tests and conditional logistic regression were used to identify comorbidities over-represented in the COPD cases compared to the control population, as appropriate. Results were reported as odds-ratio together with associated 95% confidence intervals and *p*-values. The relevant comorbidities of interest were defined as any comorbidity significantly over-represented in the COPD cases (Bonferroni-adjusted *p*-values <0.05) and with a prevalence ≥ 0.5%.

The presence/absence of relevant comorbidities in the COPD cases was compiled into a table of 0/1s and the correlations among over-represented comorbidities were investigated using principal component analysis (PCA). The diagnostic path, i.e. the time evolution of the comorbidities leading to COPD, was assessed using between-class PCA (BCA). BCA is a supervised counterpart of PCA using external information of a single categorical variable to find a low-dimensional subspace which best represents the inter-temporal variations. In the current study, time of hospitalizations prior to the onset of COPD was categorized into 2-year ranges. Inter-time BCA was used to identify the combination of comorbidities that best discriminates among time sequences.

Disease-network analyses were used to explore and visualize the directionality of the pre-diagnoses of COPD. In order to reduce the complexity of the disease-network visualization, only prevalent comorbidities involved in the current data set in at least 400 trajectories were retained. All analyses were done using the R statistical software (v. 4.0.4) including the extension packages RSQLite ADE4, vegan, comorbidity, ICD10gm and igraph.

### Validation strategy

The Danish disease trajectory browser (DTB) was used as a validation cohort [32]. The data from this population-wide cohort provides information about disease progression patterns from 7.2 million patients, encounters at Danish hospitals (in- and outpatient clinics and emergency room visits). This tool was designed to identify diagnosis pairs with statistically significant directionality which can be combined into linear disease trajectories. The validation was applied to all over-represented conditions by enumerating the number of linear disease trajectories identified in the DTB. Notice that the DTB relies on a 2-digit ICD-10 code level. For the purpose of the current validation, the over-represented comorbidities of interest were also coded using the same 2-digit hierarchical level, in order to assure the compatibility between the two databases.

## Results

### Hospitalizations preceding the onset of COPD

**Fig 1:**
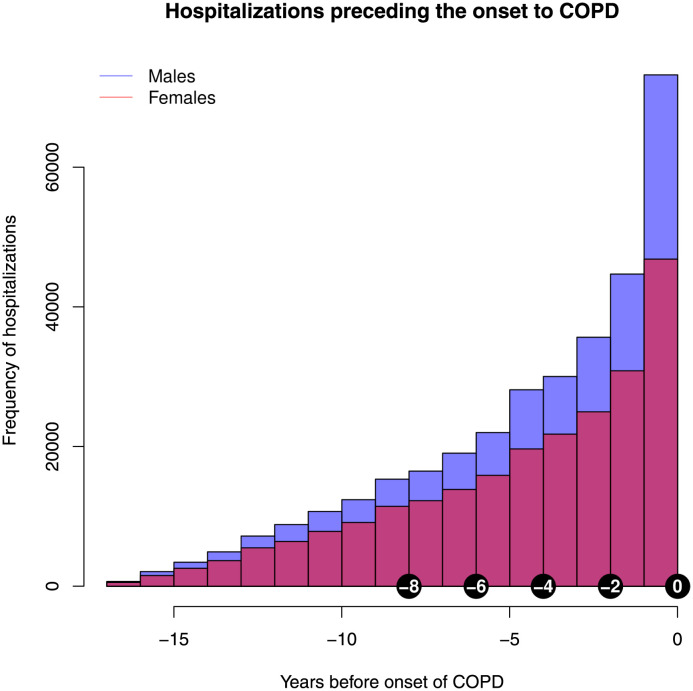
Number of hospitalizations preceding the initial diagnosis of COPD in both genders.

Between 2002 and 2018, 697,714 hospitalizations coded with a first- or co-diagnosis of COPD were recorded, corresponding to 257,164 unique patients. In 145,271 unique patients, 569,889 antecedent hospitalizations were identified ranging from 1 month until 17 years prior to COPD (Fig 1). The median number of hospitalizations per patient was 3 (IQR: 1 to 5). Fifty-nine percent of cases were males and the median age category in both genders was 65–69. The median length of stay was 5 days (IQR: 2 to 12 days) compared to 9 days (IQR: 4 to 15 days) after the first diagnosis of COPD. The median number of comorbidities was 3 (IQR: 1 to 6) in both genders compared to 8 (IQR: 5 to 10) after diagnosis of COPD.

### Conditions preceding the onset of COPD

Overall, 11,003 comorbidities were coded in the hospitalizations preceding the initial COPD diagnosis. Sixty-two were significantly over-represented in COPD compared to the controls (adjusted *p*-value <0.05 and prevalence ≥ 0.5%) (see details in Table 1).

**Table 1:** List of comorbidities significantly over-represented in the population of patients prior to COPD onset compared to a matched-control population. Rows are grouped according to the time to onset of COPD using a gradient color code from light red (former comorbidity) to dark red (recent comorbidity). Within each time category, rows are ordered according to odds-ratios. The stars next to the ICD-10-GM code indicate that the comorbidity was part of a confirmed trajectory from the Danish disease trajectory browser.

ICD-10-GM code	Description	Prevalence (%)	Odds-ratio (95% CI)
F192	Multiple drug use	0.62	4.7 (4.3 to 5.1)
F112*	Opioid dependence syndrome	0.62	4.3 (4 to 4.7)
F171*	Harmful use of tobacco	3.67	2.7 (2.6 to 2.7)
F172*	Tobacco dependence syndrome	2.85	2.5 (2.5 to 2.6)
F200	Paranoid schizophrenia	0.74	2.1 (2 to 2.2)
B182*	Chronic viral hepatitis C	0.54	1.9 (1.8 to 2)
I702*	Atherosclerosis of arteries of extremities	1.63	1.7 (1.7 to 1.8)
F331*	Recent depressive disorder	0.77	1.6 (1.5 to 1.7)
G473*	Sleep apnea	0.96	1.6 (1.5 to 1.6)
Z864	Personal history of psychoactive substance abuse	0.73	1.4 (1.3 to 1.4)
Z958	Presence of cardiac and vascular implants	0.66	1.4 (1.3 to 1.5)
E669	Obesity, unspecified	2.35	1.2 (1.1 to 1.2)
M480	Spinal stenosis	0.72	1.2 (1.1 to 1.3)
M544	Lumbago with sciatica	0.82	1.2 (1.2 to 1.3)
E660	Obesity, due to excess calories	1.06	1.1 (1.1 to 1.2)
H259	Senile cataract	1.69	1.1 (1.1 to 1.2)
F132	Dependence syndrome of sedatives	0.75	2.9 (2.7 to 3.1)
F102*	Alcohol dependence syndrome	5.00	2.8 (2.7 to 2.8)
F100*	Acute alcohol intoxication	0.91	2.3 (2.2 to 2.5)
F101*	Harmful use of alcohol	1.26	2.2 (2.1 to 2.3)
I714*	Abdominal aortic aneurysm	0.73	1.7 (1.6 to 1.8)
I739	Peripheral vascular disease	1.25	1.7 (1.6 to 1.8)
F339*	Recurrent depressive disorder	0.55	1.5 (1.4 to 1.6)
F412*	Anxiety and depressive disorder	0.94	1.4 (1.3 to 1.5)
I420	Dilated cardiomyopathy	0.57	1.3 (1.2 to 1.4)
I652	Occlusion and stenosis of carotid artery	0.73	1.3 (1.2 to 1.3)
F321	Moderate depressive episode	0.60	1.2 (1.1 to 1.2)
M545	Low back pain	1.37	1.2 (1.2 to 1.3)
M819*	Osteoporosis	0.63	1.2 (1.1 to 1.3)
I259*	Chronic ischaemic heart disease	1.74	1.1 (1.1 to 1.1)
I501	Left ventricular failure	0.69	1.1 (1.1 to 1.2)
J42	Chronic bronchitis	0.84	5.4 (5 to 5.9)
J459*	Asthma	2.87	3.9 (3.7 to 4)
I7021*	Atherosclerosis of arteries of extremities	0.66	2.3 (2.2 to 2.5)
R060	Dyspnoea	0.68	1.6 (1.5 to 1.7)
J180	Bronchopneumonia	0.60	1.5 (1.4 to 1.6)
J181	Lobar pneumonia	0.98	1.5 (1.4 to 1.6)
K703	Alcoholic cirrhosis of liver	0.73	1.4 (1.3 to 1.5)
J189	Pneumonia	1.13	1.3 (1.2 to 1.3)
F329	Depressive episode, unspecified	1.91	1.2 (1.1 to 1.2)
I110	Hypertensive heart disease with heart failure	0.68	1.2 (1.1 to 1.2)
I2522*	Myocardial infarction	0.58	1.2 (1.1 to 1.2)
I481	Persistent atrial fibrillation	0.12	1.2 (1.1 to 1.3)
I509	Heart failure	0.94	1.2 (1.1 to 1.2)
N083*	Glomerular disorders in diabetes mellitus	0.60	1.2 (1.1 to 1.3)
I258*	Chronic ischaemic heart disease	0.70	1.1 (1.1 to 1.2)
Z955	Presence of coronary angioplasty implant	2.43	1.1 (1.1 to 1.1)
I7020*	Atherosclerosis of arteries of extremities	0.84	1.9 (1.8 to 2)
C341*	Malignant neoplasm of upper lobe, bronchus or lung	0.76	1.8 (1.7 to 1.9)
Z9588	Presence of cardiac and vascular implants	0.82	1.7 (1.6 to 1.8)
G4731*	Obstructive sleep apnea	0.66	1.6 (1.5 to 1.7)
I1100	Hypertensive heart disease with heart failure	0.72	1.4 (1.3 to 1.4)
I5001	Congestive heart failure	1.05	1.3 (1.2 to 1.3)
I5014	Left ventricular failure	0.56	1.3 (1.3 to 1.4)
N183*	Chronic kidney disease	1.51	1.2 (1.2 to 1.3)
E559*	Vitamin D deficiency	1.00	1.1 (1.1 to 1.2)
E871*	Hypo-osmolality and hyponatremia	1.13	1.1 (1.1 to 1.2)
E875*	Hyperkalaemia	0.52	1.1 (1.1 to 1.2)
I1190	Hypertensive heart disease without heart failure	2.32	1.1 (1.1 to 1.2)
I2519*	Atherosclerotic heart disease	1.54	1.1 (1.1 to 1.1)
Z921	Personal history of long-term use of anticoagulants	3.00	1.1 (1.1 to 1.2)
Z950	Presence of electronic cardiac devices	1.43	1.1 (1.1 to 1.2)

**Fig 2:**
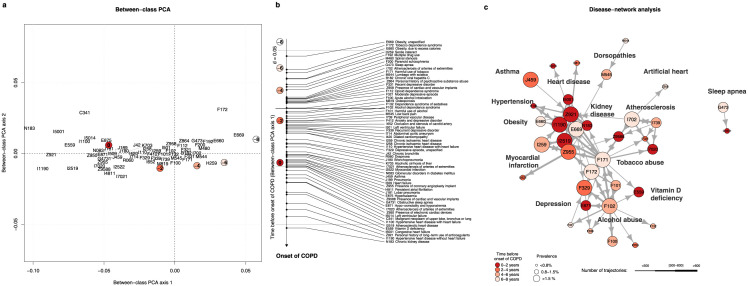
Conditions preceding the onset of COPD. Panel a shows the between-class principal component analysis (first two axes) of the time evolution of comorbidities preceding the initial COPD diagnosis. The 2-year time ranges prior to the onset of COPD are indicated by circled numbers colored from white/pale red (former comorbidities) to dark red (recent comorbidities). Preceding comorbidities are displayed using ICD-10-GM codes. The disease trajectories leading to COPD form a time gradient along the first PCA axis shown in panel b. Panel c displays the disease-network of the 36 most prevalent pre-diagnosed comorbidities involved in at least 400 trajectories in our data set. Each comorbidity is represented by a circle whose size is proportional to its prevalence and the color refers the time prior to COPD onset when the comorbidity was most prevalent. The size and the direction of the arrows joining 2 diagnoses is proportional to the number of patients diagnosed consecutively with both conditions.

Fig 2a and 2b shows the temporal evolution of the significant comorbidities prior to the onset of COPD. Diagnoses preceding the onset of COPD over 8 years included nicotine and alcohol abuse (F171, F172, F102) together with alcohol abuse-associated conditions such as hyponatremia (E871) or liver cirrhosis (K703). Mental pathologies including depression (F329, F339) and schizophrenia (F200) were also diagnosed at an early stage. Conditions over-represented 4 to 6 years before COPD diagnosis included obesity (E660, E669), sleep apnea (G473) and chronic viral hepatitis C (B182). At a later stage, conditions including chronic ischaemic heart disease (I259) and atherosclerotic heart disease (I251) were significantly over-represented. The use of artificial heart devices, implants, graft and prosthesis (Z95) were frequently observed in patients prior to developing COPD. Diagnoses of hypertensive heart disease (I119) and atherosclerosis (I702, I739) were generally identified shortly before the onset of COPD. Comorbidities occurring at a stage very close to the initial COPD diagnosis included right heart failure (I5001), vitamin D deficiency (E559), chronic kidney disease (N183) and lung cancer (C341).

Fig 2c provides an overview of the networks and directionality between the conditions preceding COPD. The flow of diagnoses includes early alcohol/tobacco abuse together with atherosclerosis followed by heart diseases (hypertensive heart disease, chronic ischaemic heart disease) ultimately leading to COPD. The following succession of conditions were associated with a high risk of short-term onset of COPD: hypertension (I10), followed by chronic ischaemic heart disease (I25) followed by the presence of cardiac pacemaker (Z95). Several pre-conditions triggered this trajectory including nicotine dependence (F17), obesity/lipidemias (E66). Intermediate diagnoses of atherosclerosis (I70) or diabetes (E11) were also identified.

### Gender-specific disease trajectories

**Fig 3:**
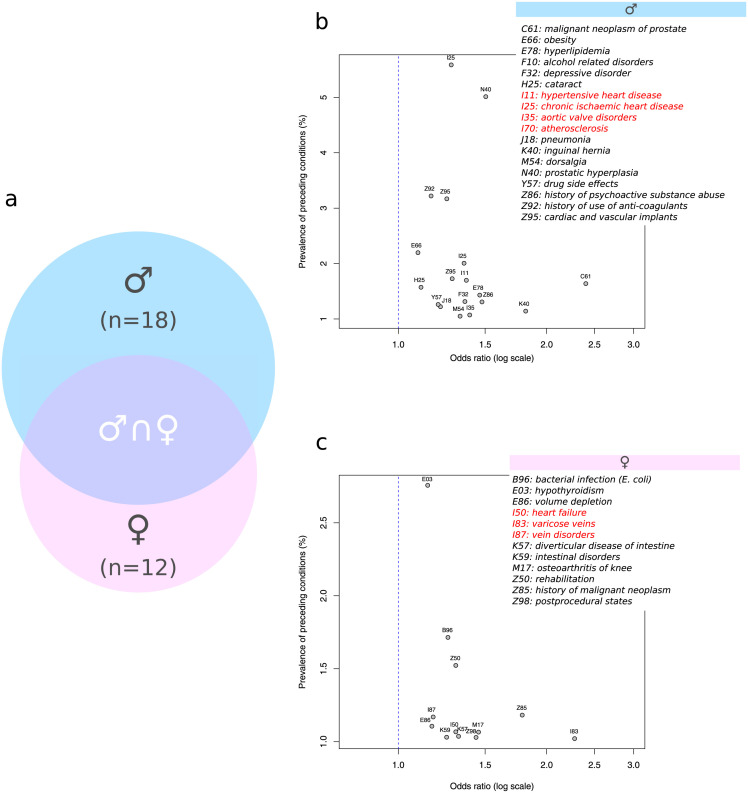
Gender-specific diagnoses preceding COPD. Panel a shows the Venn diagram of preceding conditions in males and females. Panel b shows the odds-ratio / prevalence plot of preceding conditions in males with the detailed list of ICD-10-GM codes (cardiovascular conditions are highlighted in red). Panels c shows the odds-ratio / prevalence plot of preceding conditions in females with the detailed list of ICD-10-GM codes (cardiovascular conditions are highlighted in red).

The temporal evolution of diagnoses preceding COPD (ICD-10-GM codes summarized at the 2-digit level) was separately investigated in both genders. Compared to matched controls, 18 and 12 conditions were specifically over-represented in male and female patients with COPD, respectively. Fig 3a shows the Venn diagram of the conditions specific to each gender. Conditions that were common in both genders included tobacco/nicotine dependence (F17), hypertension (I10), hypercholesterolemia (E78) and chronic kidney diseases (N18). Conditions more frequent in females included *E. coli* infections (B96), hypothyroidism (E03), heart failure (I50), varicose/venous insufficiency (I83, I87), intestinal disorders (K57, K59) and personal history of breast cancer (Z85). Conditions more frequent in males included prostate cancer (C61), obesity (E66), hyperlipidemia (E78), depressive disorder (F32), hypertensive heart disease (I11), chronic ischaemic heart disease (I25), atherosclerosis (I70), pneumonia (J18), low back pain (M54) and cardiac implant (Z95).

Fig 3b and 3c provide a gender-specific overview of the odds-ratio / prevalence of conditions preceding the onset of COPD. In males, early atherosclerotic heart diseases (I25) was highly prevalent and constituted together with hypertension (I11) important pre-conditions triggering aortocoronary bypass, angioplasty implants (Z95), and were often followed by the use long-term coagulant therapies (Z92). In females, hypothyroidism (E03) was very prevalent and was associated with heart failure (I50), vein disorders (I87), intestine disorders (K59, K57) and osteoarthritis of knee (M17).

### Independent cohort validation

Using an independent cohort available from the Danish disease trajectory browser (DTB), a total of 9,109 linear disease trajectories including COPD (J44) were identified. The most prevalent pre-diagnoses of COPD included atherosclerosis (I70) found in 1,317 cases (14%), ischaemic heart disease (I20) found in 931 cases (10%), gastritis (K29) in 722 cases (8%), and mental and behavioral disorders due to the use of alcohol (F10) found in 696 cases (8%). DTB relies on ICD-10 codes grouped at a 2-digit level. On the same hierarchical level, the 62 over-represented comorbidities were grouped into 38 2-digit ICD-10 categories. Overall 17 out of 38 comorbidities were confirmed from the DTB. The confirmed comorbidities are starred in Table 1 and more details are available in S1 Table. The most common validated conditions were atherosclerosis (I70), disorders due to psychoactive substance use (including F10, F11, F17), osteoporosis (M81) and lung cancer (C34). Other less prevalent conditions such as heart failure (I50), atrial fibrillation (I48) or pneumonia (J18) could not be directly validated from the DTB.

## Discussion

The longitudinal assessment of comorbidities recorded in the hospitalization history of patients developing COPD highlights a series of health conditions intrinsically forming distinct pathogenetic paths converging to COPD. These paths may provide early indicators of a future onset of COPD. Some of these indicators were anticipated (like nicotine and alcohol dependence and chronic viral hepatitis C) whereas others were more unexpected including obesity and atherosclerosis.

In the current study, a series of comorbidities were significantly over-represented in the years preceding the diagnosis of COPD compared to a matched control group. Conforming with the current understanding of COPD pathogenesis a clearly anticipated link between nicotine and alcohol dependence was found years before the diagnosis of COPD. The association between cigarette smoking (F17), alcohol consumption (F10), and depressive / mental disorders (F20, F32, F33, F41) is well documented in the literature [33].

Another pathophysiological link uncovered by the current analysis shows a clear association between COPD and preexisting cardiovascular entities including atherosclerosis (I70), ischaemic heart disease (I25) and hypertension (I11). The link between atherosclerosis and COPD was independently confirmed in the study from Jensen and colleagues investigating temporal disease trajectories in a health registry data set covering the whole population of Denmark [31]. They found a clear convergence between a pre-diagnosis of atherosclerosis and a subsequent COPD diagnosis.

The association between chronic viral hepatitis C (B182) and COPD has been described previously. In our study, an odds-ratio of 1.9 (95% CI: 1.8 to 2.0) was found when comparing the COPD population to the matched control population. A similar association (relative risk of 1.8) was found using the DTB. Several studies evaluated the impact of HCV infections on the development of COPD, with divergent conclusions as whether HCV is directly involved in the pathogenesis of COPD [34–39]. While most studies discussed HCV as a risk factor for the development or exacerbation of COPD, Fischer and colleagues report that HCV is not an independent risk factor for obstructive lung disease [37]. Instead, the authors concluded that there is a strong correlation between HCV status, the use of injection drugs and smoking, therefore HCV alone might not be an independent contributor to the increased prevalence of obstructive lung diseases. Inversely, other authors stated that there is a direct pathogenetic link between the two conditions. In a review, Merkov and colleagues suggested that the most likely pathogenic link between COPD and HCV is systemic inflammation [34]. HCV leads to a higher production of inflammatory mediators such TNF, IL-6 and IL-8, which play an important role in the pathogenesis of COPD. Other proposed mechanisms include cirrhosis-triggered hypertension which can affect lungs [39].

Hyponatremia (E871) is another COPD-associated comorbidity typically worsening patient’s outcome during exacerbation [40, 41]. In the current study, hyponatremia was diagnosed more often in the population developing COPD compared to the controls with an odds-ratio of 1.1 (95%CI: 1.1 to 1.2). Hyponatremia is rather associated with co-occurring cardiac pathology, e.g. atrial fibrillation and its treatment [42, 43].

Although many identified comorbidities could be validated from the DTB, a few discrepancies remained. These might be explained by specificities of the German modification of the ICD-10 codes used in the current database. Same conditions can be coded differently using congruent codes (e.g. ischaemic heart disease coded by I20 vs. I25, or depressive disorders coded as F32 or F33). Coding rules can slightly differ between the two systems (e.g. the use of codes for pneumonia vs. bacterial pneumonia). This can further explain other subtle inconsistencies. Obesity was identified in our database as a very early preceding diagnosis before the onset of COPD. The link between obesity and COPD has been described elsewhere [44, 45]. The absence of confirmed link between obesity and COPD in the Danish database might be explained by the fact that the DTB did not track back information as long as our database (14.9 years vs. 17 years).

Finally, gender-specific differences in preceding conditions were identified. A series of conditions typically associated with females such as history of breast cancer, hypothyroidism, varicose, *E. coli* infections were identified. Somewhat surprisingly, heart failure was also more common in women as a preceding condition. Overall, the incidence of heart failure is lower in women than in men. Still, it remains possible that there is a common predisposition for heart failure and COPD specifically in women, e.g. via inflammatory pathways leading to both endothelial dysfunction (and heart failure with preserved ejection fraction even without disease of the epicardial coronary arteries) and on bronchial inflammation. In contrast, usual male conditions included prostate cancer and prostatic hyperplasia. However, cardiovascular diseases were also found in particular coronary artery disease. The latter is much more common in men, at least in part mediated by sex hormones and cigarette smoking. The higher prevalence of smoking in men in combination with a common susceptibility to develop atherosclerosis and bronchial destruction may represent the basis for the association between coronary artery disease and COPD in men. Depression and anxiety contribute to the burden of COPD-related morbidity [46]. Our data showed that anxiety-related disorders were more frequently diagnosed in females, whereas depression was more often diagnosed in the male population. Literature findings previously reported a higher level of both anxiety and depression in the female population [47, 48]. Thyroid gland dysfunction had been described as a comorbid condition in COPD [49], with possible higher prevalence in females compared to males [50].

Our study has several limitations. The current study is in essence retrospective. The data analyses are based on hospitalizations occurring prior to the onset of COPD and our population derives from patients with a coded COPD. The correctness of the COPD diagnosis depends on the coding skills of the healthcare worker which may impact on the completeness of the identified COPD cases. In addition, detailed COPD-related diagnostic information was not available in the current hospitalization database.

## Conclusion

By investigating the hospitalization antecedents preceding the onset of COPD, the current study enumerates a series of comorbidities, which define distinct pathogenetic paths consequently leading to COPD. The main highlight of the current study is that distinct gender-specific trajectories could be identified reflecting differential patho-mechanisms involved in females and males. Cardiovascular diseases were frequently diagnosed prior to the onset of COPD with heart failures and vein disorders constituting an early COPD indicator in females whereas vascular and artherosclerotic diseases constituted an early COPD indicator in males. Finally, the temporal evolution of these complex disease networks provide indicators of a future onset of COPD, and as such should be carefully scrutinized in the standard clinical check-up in individuals at risk for the development of COPD.

## Supporting information

S1 Table Validation of over-represented comorbidities using the Danish disease trajectory browser. The conditions are ranked according to the number of validated trajectories.
(PDF)

## Abbreviations

BCA: Between-class principal component analysis
CI: Confidence interval
COPD: Chronic obstructive pulmonary disease
DTB: Danish disease trajectory browser
FEV_1_: Forced expiratory volume in 1 second
HCV: Hepatitis C virus
HR: Hazard ratio
ICD-10-GM: International Classification of disease, revision 10, German modification
IL-6: Interleukin 6
IL-8: Interleukin 8
IQR: Inter-quartile range
PCA: Principal component analysis
SQL: Structured query language
TNF: Tumor necrosis factor
