# Supplementary material for: Gender-specific disease trajectories prior to the onset of COPD allow individualized screening and early intervention
Source: PLoS One. 2023 Jul 7;18(7):e0288237. doi: 10.1371/journal.pone.0288237 (PMC10328310; doi:10.1371/journal.pone.0288237)
Supplement: S1 Table — The conditions are ranked according to the number of validated trajectories. (PDF) [file pone.0288237.s001.pdf]

Gender-specific disease trajectories descry pathogenetic traits in COPD allowing for individualized screening and early intervention

Michelle Hagmann, Florent Baty, Micha T Maeder, Frank Rassouli, Martin H Brutsche

Supplementary Data

eTable 1

Validation of over-represented comorbidities using the Danish disease trajectory browser. The conditions are ranked according to the number of validated trajectories.

ICD-10 (2 digits)	Description	Number of validated trajectories
I70	Atherosclerosis	1317
F10	Alcohol related disorders	813
M81	Osteoporosis	655
C34	Malignant neoplasm of bronchus and lung	551
E87	Hyponatremia/hyperkalaemia	231
N18	Chronic kidney disease	218
F41	Anxiety disorder	145
G47	Sleep disorders	97
F33	Major depressive disorder (recurrent)	92
F11	Opioid related disorders	72
E55	Vitamin D deficiency	57
B18	Chronic viral hepatitis	51
F17	Nicotine dependence	21
I71	Aortic aneurysm and dissection	14
J45	Asthma	11
N08	Glomerular disorders	5
I25	Chronic ischaemic heart disease	3
H25	Age-related cataract	1
E66	Overweight and obesity	0
F13	Sedative, hypnotic, or anxiolytic related disorders	0
F19	Psychoactive substance related disorders	0
F20	Schizophrenia	0
F32	Major depressive disorder (single)	0
I11	Hypertensive heart disease	0
I42	Cardiomyopathy	0
I48	Atrial fibrillation	0
I50	Heart failure	0
I65	Occlusion and stenosis of precerebral artery	0
I73	Peripheral vascular disease	0
J18	Pneumonia	0
J42	Chronic bronchitis	0
K70	Alcoholic liver disease	0
M48	Spondylopathies	0
M54	Dorsalgia	0
R06	Dyspnea	0
Z86	Personal history of psychoactive substance abuse	0
Z92	Personal history of long-term use of anticoagulants	0
Z95	Presence of cardiac and vascular implants and grafts	0
